# HLA‐C*06:02 allele can influence clinical efficacy of certolizumab pegol?

**DOI:** 10.1002/hsr2.234

**Published:** 2021-05-04

**Authors:** Annunziata Dattola, Elisabetta Botti, Marco Galluzzo, Dante Raffaele Caro Caposiena, Sara Mazzilli, Elena Campione, Gennaro Melino, Eleonora Candi, Luca Bianchi

**Affiliations:** ^1^ Department of Dermatology University of Rome “Tor Vergata” Rome Italy; ^2^ Department of Experimental Medicine University of Rome “Tor Vergata” Rome Italy

## 1 INTRODUCTION

Psoriasis is a chronic inflammatory skin disease characterized by erythematous plaques with scales mainly localized at the elbows, knees, and the trunk, but, which can affect all skin areas including the scalp or genital area. To the date, many drugs are developed to treat this condition, in particular biological treatments are the most important therapy that are used to control the disease.

We read with very interesting the paper published on January 16 by Sin et al^1^ regarding the role of the Human Leukocyte Antigen C06 (HLA‐C*06) in Chinese patients with active peripheral type psoriatic arthritis. The authors enrolled 60 patients but only 47 patients were genotyping for HLA‐C*06. The result showed HLA‐C*07:02 was the most frequent allele (29.8%), followed by HLA‐C*01 (26.6%). The frequency of HLA‐C*06:02 alleles was similar to Chinese normal population. We present data from our study conducted in patients with psoriatic arthritis (PsA) and chronic plaque psoriasis treated with certolizumab pegol and despite previous methotrexate, cyclosporine, anti‐inflammatory drugs (NSAIDs), and corticosteroids; other authors reported similar data in response to adalimumab and ustekinumab.^2^, ^3^

## 2 METHODS

We reviewed data of 39 Caucasian patients genotyped for HLA‐C*06, 18 out of 39 patients were naive to biologic treatment, all patients were evaluated by both rheumatologists and dermatologists for the diagnosis. In our cohort, 19 patients were male and 20 females, the mean age was 56.0 ± 12.1 years (mean ± SD) and the duration of the PsA disease was 6.7 ± 5.4 years (Table 1).

**TABLE 1:** Demographic and clinical characteristics of patients at baseline

Data		HLA‐C*06 +	HLA‐C*06 −	Total
Gender	Male	4 (40%)	15 (52%)	19 (48.7%)
	Female	6 (60%)	14 (48%)	20 (51.3%)
Age (years ± SD)		54.3 ± 10.5	56.6 ± 12.6	56.0 ± 12.1
Smoke (yes/no)		1 (10%)	5 (17%)	6 (15.4%)
Alcohol intake (yes/no)		3 (30%)	4 (14%)	17 (43.6%)
BMI (±SD)		27.8 ± 27.8	29.3 ± 3.9	28.9 ± 4.0
Body weight (kg ± SD)		74.9 ± 9.9	80.9 ± 10.8	79.4 ± 10.9
Disease duration (years ± SD)	PsO	8.9 ± 6.7	10.0 ± 10.0	9.7 ± 9.3
	PsA	7.8 ± 6.8	6.3 ± 4.7	6.7 ± 5.4
Naïve patients (yes/no)		7 (70%)	11 (37.9%)	18 (46.2%)

Abbreviation: PsA, psoriatic arthritis; PsO, psoriasis.

HLA‐C*06:02 genotyping was performed on DNA samples of all 39 subjects; venous blood for genotyping was collected in ethylenediaminetetraacetic acid tubes and stored at −70°C. DNA was extracted from whole blood using DNeasy Blood & Tissue kit (Quiagen, Hilden, Germany). The HLA‐C*06 allele was detected by standard polymerase chain reaction using allele specific primers (forward 5′‐TACTACAACCAGAGCGAGGA‐3′; reverse 5′‐GGTCGCAGCCATACATCCA‐3′). Ten out of 39 (26%) were HLA‐C*06 positive (six in heterozygosis and four in homozygosis).

The severity of psoriasis and response to treatment were evaluated using the Psoriasis Area and Severity Index (PASI) score at baseline and then at follow‐up visits on weeks 12, 24, and 52. We evaluated the proportion of patients achieving ≥50% reduction in PASI score (PASI 50), a ≥75% reduction in PASI score (PASI 75), a ≥90% reduction in the PASI score (PASI 90), and a ≥100% reduction in the PASI score after 12, 24, and 52 weeks. We evaluated also the following variables: Disease Activity Score (DAS‐44), Patients Visual Analogue Scale (VAS), Modified Nail Psoriasis Severity Index (mNAPSI), Dermatology Life Quality Index (DLQI), Tender Joint Count (TJC), Swollen Joint Count (SJC), C‐Reactive Protein (CRP), and Erythrocyte Sedimentation Rate (ESR) at each time point.

## 3 RESULTS

An ANOVA analysis was realized and no differences statistically significant were found between HLA‐C*06 positive and negative patients at baseline. Additionally, no correlations were found between clinical response and presence of allele C*06 at univariate logistical regression analysis; although a better clinical response at week 12, defined by PASI‐50 (coef [CI] = −1.98 [0.03‐0.99] *P*‐value = .048), PASI‐100 (coef [CI] = −2.75 [0.27‐0.80] *P*‐value = .006), DAS‐44 (coef [CI] = −2.52 [0.29‐0.86] *P*‐value = .012), and at week 52, defined by DAS‐44 (coef [CI] = −2.15 [0.70‐0.98] *P*‐value = .031), was correlated with the number of biological treatments used. Furthermore, a correlation between body weight and clinical outcome at week 12, defined by DAS‐44 (coef [CI] = −2.23 [0.69‐0.98] *P*‐value = .026), and ESR level at week 24 (coef [CI] = −2.61 [0.45‐0.98] *P*‐value = .009) were observed (Table 2).

**TABLE 2:** Patients' disease score

	Baseline		Week 12		Week 24		Week 52	
	HLA‐C*06 +	HLA‐C*06 −	HLA‐C*06 +	HLA‐C*06 −	HLA‐C*06 +	HLA‐C*06 −	HLA‐C*06 +	HLA‐C*06 −
PASI	4.8 ± 1.6	4.7 ± 4.5	2.2 ± 1.7	2.8 ± 4.0	1.3 ± 1.9	0.9 ± 2.0	0.75 ± 1.4	0.8 ± 2.1
mNAPSI	19.3 ± 6.6	15.0 ± 7.0	5.5 ± 3.3	6.0 ± 1.6	2.0 ± 1.6	2.7 ± 1.9	0.0 ± 0.0	2.0 ± 1.6
TJC	11.7 ± 7.1	9.59 ± 6.3	5 ± 3.8	3.9 ± 2.9	1.1 ± 1.8	1.2 ± 1.4	1.1 ± 2.0	0.9 ± 2.0
SJC	5.3 ± 4.4	5.4 ± 4.0	2.1 ± 2.6	1.9 ± 2.0	0.3 ± 0.6	0.7 ± 1.6	0.3 ± 0.7	0.5 ± 1.3
CRP	3.9 ± 5.5	4.6 ± 8.6	1.0 ± 0.0	0.9 ± 0.3	0.8 ± 0.4	0.8 ± 0.4	0.9 ± 0.3	0.8 ± 0.4
ESR	25.1 ± 13.2	21.6 ± 16.5	0.6 ± 4.9	0.9 ± 0.3	0.8 ± 0.4	0.8 ± 0.4	0.6 ± 0.5	0.6 ± 0.5
Pain VAS	68.0 ± 15.4	70.5 ± 19.2	23.0 ± 19	35.9 ± 24.6	22.0 ± 20.9	19.2 ± 17.7	10.0 ± 10.0	18.1 ± 18.4
DLQI	16.7 ± 5.3	19.0 ± 4.8	7.3 ± 2.2	9.7 ± 4.9	6.3 ± 2.4	5.9 ± 3.4	5.38 ± 2.9	4.7 ± 3.9
DAS‐44	3.5 ± 0.9	3.3 ± 0.8	0.8 ± 0.4	0.7 ± 0.5	0.9 ± 0.3	1.0 ± 0.2	1.0 ± 0.0	1.0 ± 0.2

Abbreviations: CRP, C‐Reactive Protein; DAS‐44, Disease Activity Score; DLQI, Dermatology Life Quality Index; ESR, Erythrocyte Sedimentation Rate; mNAPSI, Modified Nail Psoriasis Severity Index; Pain VAS, Patients Visual Analogue Scale; PASI, Psoriasis Area and Severity Index; SJC, Swollen Joint Count; TJC, Tender Joint Count.

## 4 DISCUSSION

Our data suggest that only 26% of patients affected by psoriatic arthritis are carriers of the HLA‐C*06 allele, in line with the data reported by Sin et al. Furthermore, HLA‐C*06 allele seems not correlated with a higher disease severity or a better clinical outcome. On the other hand, a multiple biological treatment failure and a high heavy weight seem to be connected with a poor response to certolizumab pegol.

## CONFLICT OF INTEREST

The authors declare no conflicts of interest.

## AUTHOR CONTRIBUTIONS

Conceptualization: Annunziata Dattola, Elisabetta Botti

Writing ‐ original draft preparation: Annunziata Dattola, Dante Raffaele Caro Caposiena

Writing ‐ review and editing: Marco Galluzzo, Sara Mazzilli, Elena Campione, Gennaro Melino, Eleonora Candi, Luca Bianchi, Annunziata Dattola
